# Supplementary material for: What does it take to learn the rules of RNA base pairing? A lot less than you may think
Source: bioRxiv. 2025 Aug 2:2025.07.31.668042. Preprint. [Version 1] doi: 10.1101/2025.07.31.668042 (PMC12324431; doi:10.1101/2025.07.31.668042)
Supplement: Supplement 1 [file media-1.gz › supplemental_material/src/rscape_v2.6.0.tar.gz!/rscape_v2.6.0/lib/hmmer/documentation/userguide/inclusions/HMMD_SEARCH_STATS.pdf]

Elapsed (8 bytes)	User (8 bytes)
Sys (8 bytes)	Z (8 bytes)
DomZ (8 bytes)	Z_setby (1 byte)
DomZ_setby (1 byte)	Nmodels (8 bytes)
Nnodes (8 bytes)	Nseqs (8 bytes)
Nres (8 bytes)	N_past_msv (8 bytes)
N_past_bias (8 bytes)	N_past_vit (8 bytes)
N_past_fwd (8 bytes)	Nhits (8 bytes)
Nreported (8 bytes)	Nincluded (8 bytes)
Hit_offsets (nhits * 8 bytes)	
